# Fluocinolone acetonide vitreous insert for chronic diabetic macular oedema: a systematic review with meta-analysis of real-world experience

**DOI:** 10.1038/s41598-021-84362-y

**Published:** 2021-02-26

**Authors:** Matteo Fallico, Andrea Maugeri, Andrew Lotery, Antonio Longo, Vincenza Bonfiglio, Andrea Russo, Teresio Avitabile, Claudio Furino, Gilda Cennamo, Martina Barchitta, Antonella Agodi, Paola Marolo, Luca Ventre, Paolo Caselgrandi, Michele Reibaldi

**Affiliations:** 1 Department of Ophthalmology, University of Catania, Via S. Sofia 78, 95123 Catania, Italy; 2 Department of Medical and Surgical Sciences and Advanced Technologies “GF Ingrassia”, University of Catania, Via S. Sofia 87, 95123 Catania, Italy; 3 Faculty of Medicine, University of Southampton, Southampton, UK; 4 Department of Experimental Biomedicine and Clinical Neuroscience, Ophthalmology Section, University of Palermo, 90127 Palermo, Italy; 5 Department of Ophthalmology, University of Bari, 70124 Bari, Italy; 6 Department of Public Health, University of Naples Federico II, 80131 Naples, Italy; 7 Department of Surgical Sciences, Eye Clinic Section, University of Turin, 10122 Turin, Italy

**Keywords:** Diabetes complications, Retinal diseases

## Abstract

We conducted a meta-analysis of real-world studies on the 0.19 mg Fluocinolone Acetonide (FAc) intravitreal implant for chronic diabetic macular oedema (DMO), comparing these findings with the Fluocinolone Acetonide for Diabetic Macular Edema (FAME) study. The primary outcome was mean change of best corrected visual acuity (BCVA) at 24 months. Secondary outcomes were 36-month mean BCVA, mean central macular thickness (CMT) change, rates of eyes receiving supplementary intravitreal therapy, cataract surgery, intraocular pressure (IOP)-lowering drops and glaucoma surgery. Mean differences (MDs) with 95% confidence intervals (CIs) were calculated. Nine real-world studies were included. The FAc implant yielded a significantly improved BCVA at 24 and 36 months (24-month MD = 4.52; 95% CI 2.56–6.48; 36-month MD = 8.10; 95% CI 6.34–9.86). These findings were comparable with the FAME study. The FAc implant yielded significantly reduced 24- and 36-month CMT. Pooled proportions of cataract surgery, IOP-lowering drops and glaucoma surgery were 39%, 27% and 3%, respectively, all lower than the FAME study. Pooled estimate of supplementary intravitreal therapy was 39%, higher than the 15.2% of the FAME study. This meta-analysis of real-world studies confirms favorable visual and anatomical outcomes following FAc insert for chronic DMO. In real-life studies more than one third of patients received supplementary intravitreal therapy, an issue that needs to be further explored.

## Introduction

Diabetic macular oedema (DMO) is a major cause of visual loss in working age people of developed countries^1^. Remarkable improvements have been made in DMO treatment over the last few decades, thanks to the introduction of intravitreal therapy with anti-Vascular Endothelium Growth Factor (anti-VEGF) agents and corticosteroids^2^. Despite such breakthroughs, a consistent percentage of patients develops a chronic persistent DMO, which has been reported as high as 55% of cases after 2 years of treatment^3^. Additionally, in real life, patients receiving anti-VEGF therapy are undertreated with worse functional outcomes compared with randomized controlled trials^4^.

The 0.19 mg Fluocinolone Acetonide (FAc) intravitreal implant (ILUVIEN), releasing on average 0.2 µg/day for up to 36 months, has been approved for the treatment of chronic DMO that is not sufficiently responsive to available therapies^5^.

The effectiveness of this long-acting implant in DMO patients has been demonstrated in the Fluocinolone Acetonide for Diabetic Macular Edema (FAME) studies A and B. These were two parallel, multicenter, 36-month randomized clinical trials (RCTs)^6^. A visual gain ≥ 15 letters was found in 28.7% of patients treated with the 0.2 µg/day FAc implant at 24 months^7^, and was maintained at 36 months^6^. Cataract progression was recorded in more than 80% of phakic eyes over the study period, while incisional glaucoma surgery was needed in 4.8% of patients due to high intraocular pressure (IOP)^6^.

Following the FAME study, many other studies have provided real-world data on the 0.2 µg/day FAc implant in patients with chronic DMO^8–17^. Real-world findings can be considered a reliable indicator of clinical practice, often being different from the evidence reported by RCTs^4^.

The purpose of this systematic review with meta-analysis of real-world studies on 0.2 µg/day FAc intravitreal implant in chronic DMO is to provide a complete picture of long-term outcomes of the implant in clinical practice and to assess whether these findings overlap with those reported by the FAME study.

## Results

**Figure 1:**
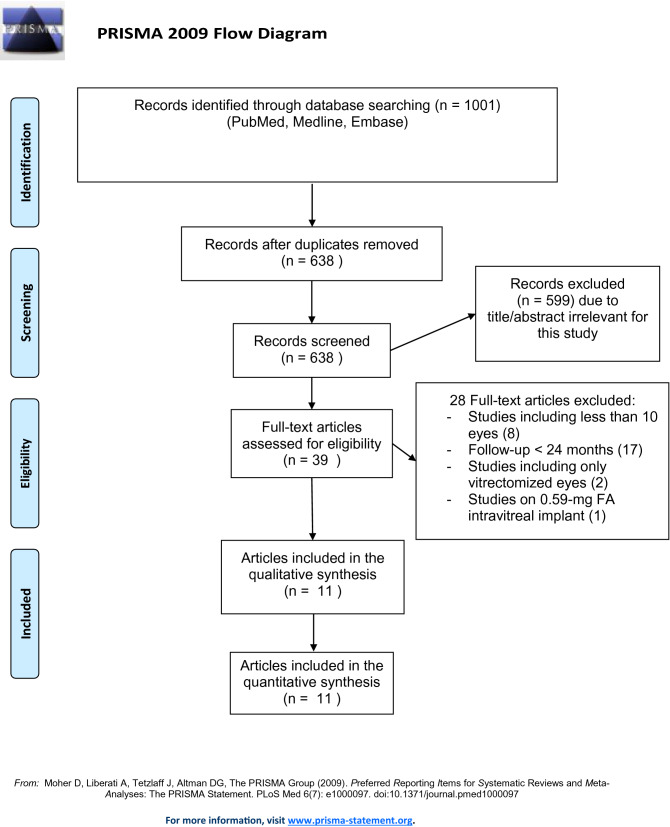
Flow chart of study selection process.

Figure 1 shows the study selection process. A total of 1001 articles were identified. Following removal of duplicates, 638 articles were screened, of which 39 articles were deemed potentially eligible and were full-text evaluated. A total of 11 articles met eligibility criteria and were included, of which two reported the 24- and 36-month result of the same RCT, the FAME study^6,7^.

### Study characteristics

A total of 9 real-world studies^8–16^ and one RCT^6,7^ were included in this systematic review. The only RCT was the FAME study, which consisted of two parallel, multicenter, 36-month randomized clinical trials comparing the 0.2 µg/day FAc implant, the 0.5 µg/day FAc implant and a sham. A total of 375 eyes were enrolled in the 0.2 µg/day FAc implant group, of which 301 and 270 eyes were included in the 24-month and 36-month analyses, respectively^6,7^. Patient enrollment started in 2007 and the study was completed in 2010. The 24-month and 36-month results were published in 2011 and 2012, respectively. Foveal thickness evaluation was based on a time-domain OCT, namely the Stratus 3 OCT instrument. Baseline phakic eyes were 235 in the 0.2 µg/day FAc implant group^6,7^.

The 9 real-world studies consisted of 7 retrospective^9–11,13–16^ and 2 prospective reports^8,12^. Publication year ranged from 2017 to 2020^8–16^. All studies reported the 24-month visual outcome, with a total of 428 eyes included in this analysis^8–16^. Six out of the 9 studies reported the 36-month visual outcome, with a total of 102 eyes^9,10,13–16^. Seven of the 9 studies provided information on the 24-month CMT outcome^8–10,13–16^, while 6 out of the 9 studies provided the 36-month CMT^9,10,13–16^. Bailey et al. provided CMT change at the last observation, which was considered within the 24-month analysis^11^. All studies but one specified that OCT imaging was based on the use of spectral domain-OCT instruments^8–10,13–16^. Bailey et al. did not provide this information^11^. Mean follow-up ranged from 14.1 to 36 months^8–16^. Three out of the 9 studies included only pseudophakic eyes^9,10,13^, of the remaining 6 reports, 3 provided information of how many phakic patients underwent cataract surgery^8,14,16^. Eight out of the 9 studies reported information on how many eyes received supplementary intravitreal therapy throughout the follow-up^8–12,14–16^. Data on type of drug, mean number of injections, and mean time from FAc implant are shown in Table 1. Eight out of the 9 studies reported information on the number of eyes that had received macular laser, either focal or grid, before FAc implant^9–16^^.^ Information on panretinal photocoagulation (PRP) treatment delivered before FAc implant was provided by 4 out of the 9 studies^12–14,16^. Following FAc implant, 5 studies^10–12,15,16^ reported data on eyes receiving additional laser treatment, either macular or PRP (Table 2).

**Table 1:** Supplementary intravitreal therapy after FAc implant of included studies.

Author, year	Number of eyes with supplementary intravitreal therapy	Drug (number of eyes)	Injection number (mean)	Mean time from FAc
Panos et al.^9^	13 out of 24	Aflibercept (4) Ranibizumab (8) Triamcinolone (2) DEX implant (1)	Aflibercept (5.8) Ranibizumab (5.1) Triamcinolone (3.5) DEX implant (1)	13.5 months
Fusi-Rubiano et al.^10^	18 out of 29	Aflibercept (11) Bevacizumab (4) Ranibizumab (3) Triamcinolone (3)	Overall, a mean of 2.6 injections	12 months
Augustin et al.^16^	25 out of 81	Aflibercept (20) Bevacizumab (8) Ranibizumab (7) DEX implant (7)	Aflibercept (3.8) Bevacizumab (2.3) Ranibizumab (1.9) DEX implant (1)	n.r
Chakravarthy et al.^12^	172 out of 593	Anti-VEGF (133) Steroid (39)	Anti-VEGF (5) Steroid (1.9)	356 days
Bailey et al.^11^	111 out of 345	Aflibercept (47) Bevacizumab (4) Ranibizumab (61) Triamcinolone (8) DEX implant (8)	Aflibercept (4) Bevacizumab (2.5) Ranibizumab (4.5) Triamcinolone (1.3)	n.r
Rehak et al.^14^	17 out of 49	Anti-VEGF (6) DEX implant (13)	Anti-VEGF (2) DEX implant (1.5)	22.2 months
Young et al.^15^	5 out of 21	Anti-VEGF (5)	Anti-VEGF (12.2)	n.r
Mansour et a.l^8^	65 out of 115	n.r	n.r	n.r
Ahmed et al.^13^	n.r	n.r	n.r	n.r

*FAc* fluocinolone acetonide, *anti-VEGF* anti-Vascular Endothelial Growth Factor, *DEX* dexamethasone, *n.r.* not reported.

**Table 2:** Laser treatment before and after FAc implant of included studies.

Author, year	Number of eyes at the baseline	Before Fac, number of eyes		After FAc, number of eyes	
		Macular laser	PRP	Macular laser	PRP
Panos et al.^9^	24	18	n.r	n.r	n.r
Fusi-Rubiano et al.^10^	29	10	n.r	4	n.r
Augustin et al.^16^	81	45	55	14 (either focal or PRP)	
Chakravarthy et al.^12^	593	181	192	57 (thermal laser)	
Bailey et al.^11^	345	98	n.r	22	n.r
Rehak et al.^14^	49	21	17	n.r	n.r
Young et al.^15^	21	17	n.r	2	n.r
Mansour et al.^8^	115	n.r	n.r	n.r	n.r
Ahmed et al.^13^	26	7	5	n.r	n.r

*FAc* fluocinolone acetonide, *PRP* panretinal photocoagulation, *n.r*. not reported.

All studies reported the proportion of both eyes receiving IOP-lowering drops and eyes undergoing glaucoma surgery^8–16^. The proportion of eyes receiving IOP-lowering drops ranged from 7 to 46%^8–16^. Chakravarthy et al. reported the use of IOP-lowering medications in 138 out of 593 eyes (23%), of which 62 received monotherapy, 30 received 2 medications, 21 received 3 medications, 35 received more than 3 medications^12^. Time of ocular hypertension onset was recorded by Fusi-Rubiano et al.: an IOP higher than 27 mmHg was reported in 2 eyes out of 29 (7%), occurring in one case at one month and in the other at six months from FAc implant^10^. Mean IOP change throughout the follow-up was shown in 4 studies^8,9,11,16^. Mansour et al. reported a mean IOP of 14.9 mmHg, 16.8 mmHg and 15.8 mmHg at baseline, 1 year and 2 years, respectively^8^. Panos et al. showed that median IOP was 16 mmHg, 18 mmHg and 17 mmHg at baseline, 1 year and 2 years, respectively^9^. In the study of Augustin et al., mean IOP changed from 15.8 mmHg at baseline to 18.2 mmHg, 15.7 mmHg and 15.6 mmHg at 1 year, 2 years and 3 years, respectively^16^. Bailey et al. reported a mean IOP of 15.7 mmHg at baseline, increasing to 19.2 mmHg and 18.3 mmHg at 1 and 2 years, respectively, reducing to 15.4 mmHg at 30 months^11^. No eye underwent glaucoma surgery in 3 studies^9,10,14^. Of the remaining 6 studies, 4 studies reported glaucoma surgery in 2–4% of cases^8,12,13,16^, while Bailey et al.^11^ reported glaucoma surgery in 0.3% of cases and Young et al.^15^ in 9.5% of cases. Chakravarthy et al. recorded mean time from FAc implant to glaucoma surgery as 8 months^12^; glaucoma surgery was performed between 24 and 30 months after the implant in the report of Ahmed et al.^13^.

Fusi-Rubiano et al. included 3 eyes that had undergone prior vitrectomy, of which one reached 24-month follow-up^10^. Young et al. included one eye that had received prior vitrectomy^15^. Augustin et al. included 39 out of 81 eyes that had been previously vitrectomized^16^. An additional 0.2 µg/day FAc implant was administered in one eye (2% of cases) in the study by Rehak et al.^14^, in 4 eyes (8.6% of cases) in the study by Augustin et al.^16^ and in 6 eyes (1% of cases) in the study by Chakravarthy et al.^12^ In the FAME study 50 patients (13.3% of cases) received additional 0.2 µg/day FAc inserts over a 36-month follow-up^6^.

### Risk of bias assessment

All real-world studies were given a MINORS score ≥ 11 (Supplementary Table S1 online). Funnel plots inspection revealed a nearly symmetrical shape for each outcome explored. Egger’s test confirmed no significant publication bias (Supplementary Fig. S1-4 online).

### Visual outcome

**Figure 2:**
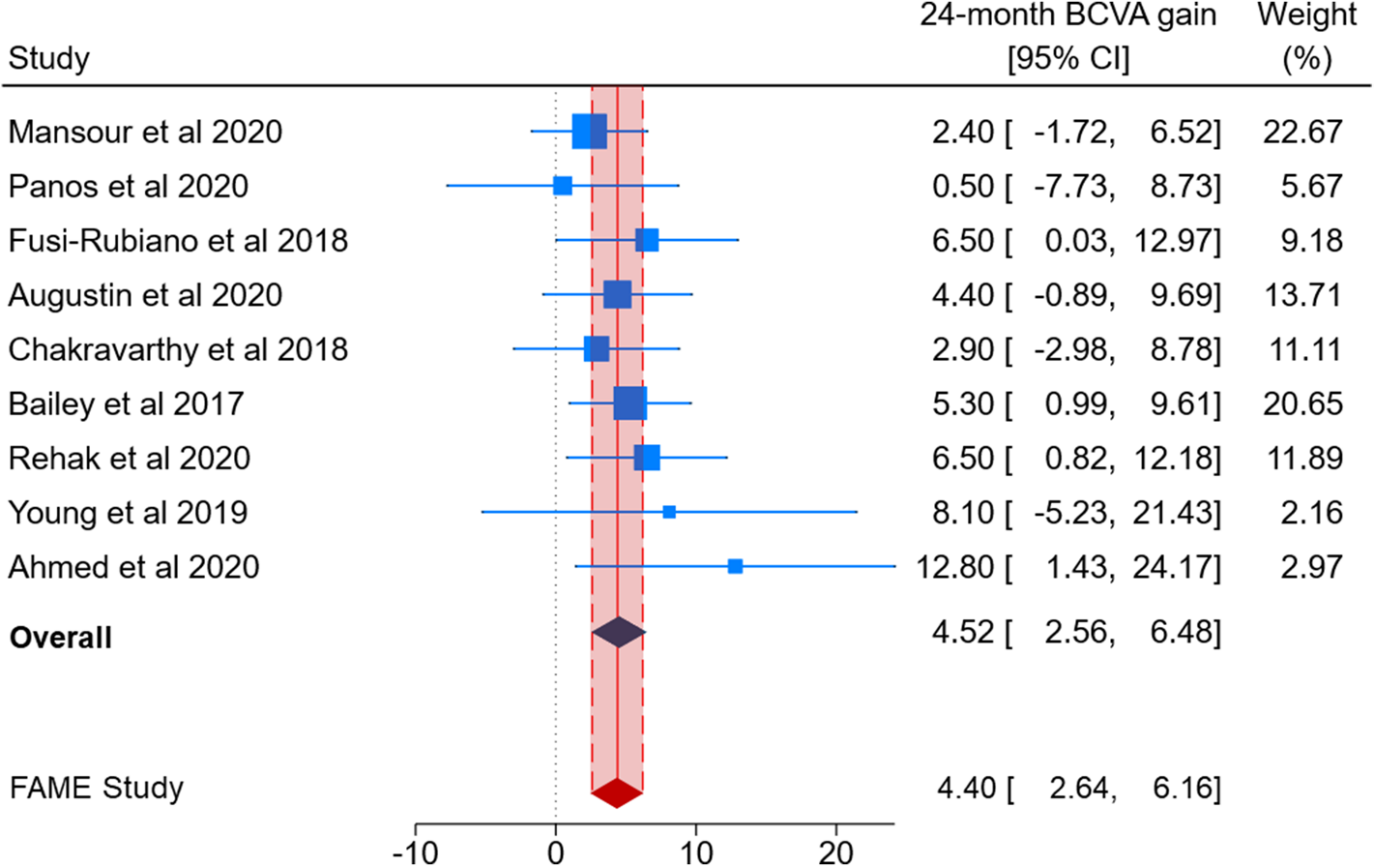
A forest plot showing 24-month best corrected visual acuity (BCVA) gain, reported as Mean Difference (MD) with 95% confidence interval (CI), in real-world studies and 24-month BCVA gain in the FAME study.

**Figure 3:**
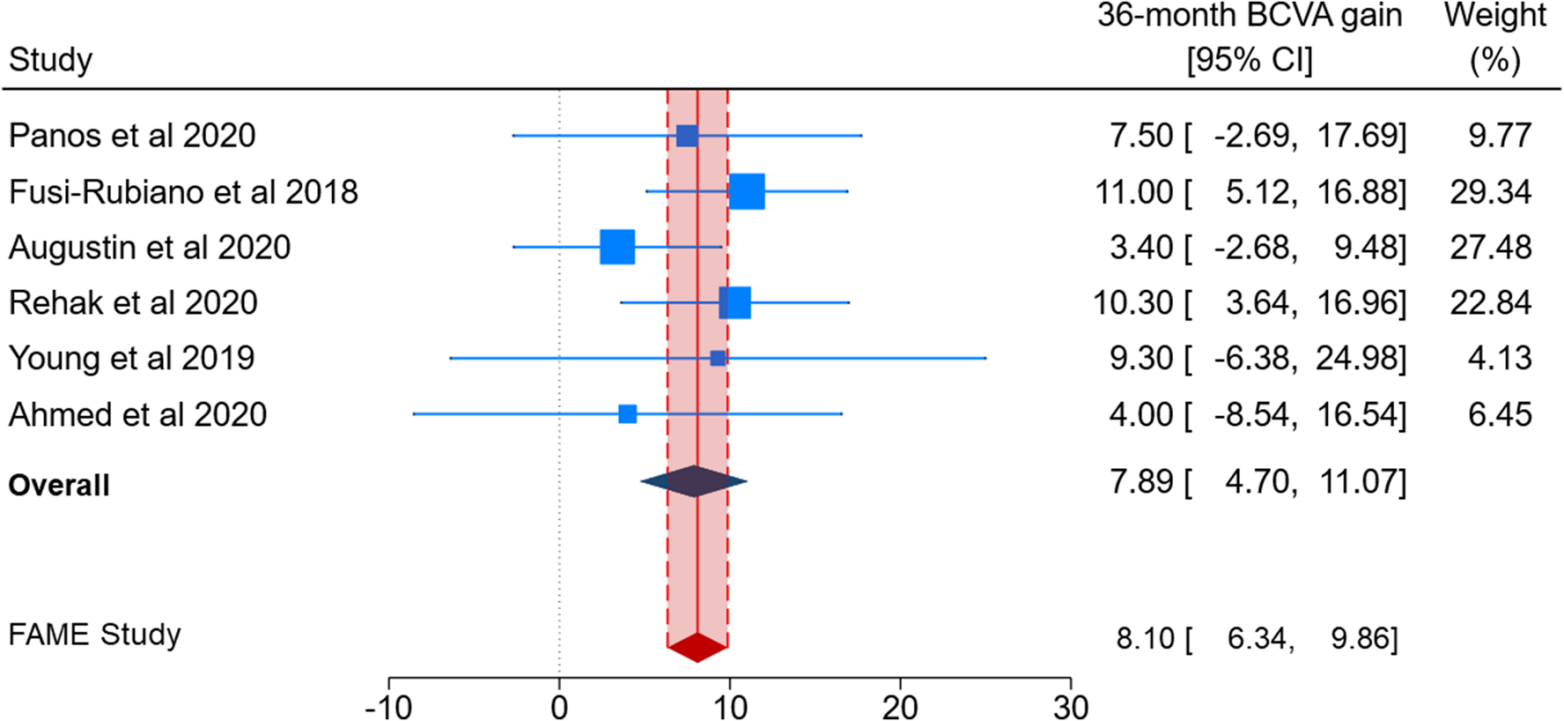
A forest plot showing 36-month best corrected visual acuity (BCVA) gain, reported as Mean Difference (MD) with 95% confidence interval (CI), in real-world studies and 36-month BCVA gain in the FAME study.

Figures 2 and 3 show the comparison of BCVA change obtained by pooling real-world studies with that reported by the FAME study. The 0.2 µg/day FAc implant yielded a significantly improved BCVA at the 24-month follow-up in nine real-world studies (MD = 4.52; 95% CI 2.56–6.48; Fig. 2). A similar result was obtained by pooling six real-world studies reporting BCVA change at the 36-month follow-up (MD = 8.10; 95% CI 6.34–9.86; Fig. 3). In both cases, no significant heterogeneity across studies was evident (*p* values for Q-statistics > 0.1 and I^2^ = 0%). Although effect sizes for some individual studies fell outside the 95% CIs reported by the FAME study (i.e. red diamonds in Figs. 2, 3), pooled estimates were comparable.

### Central macular thickness change

**Figure 4:**
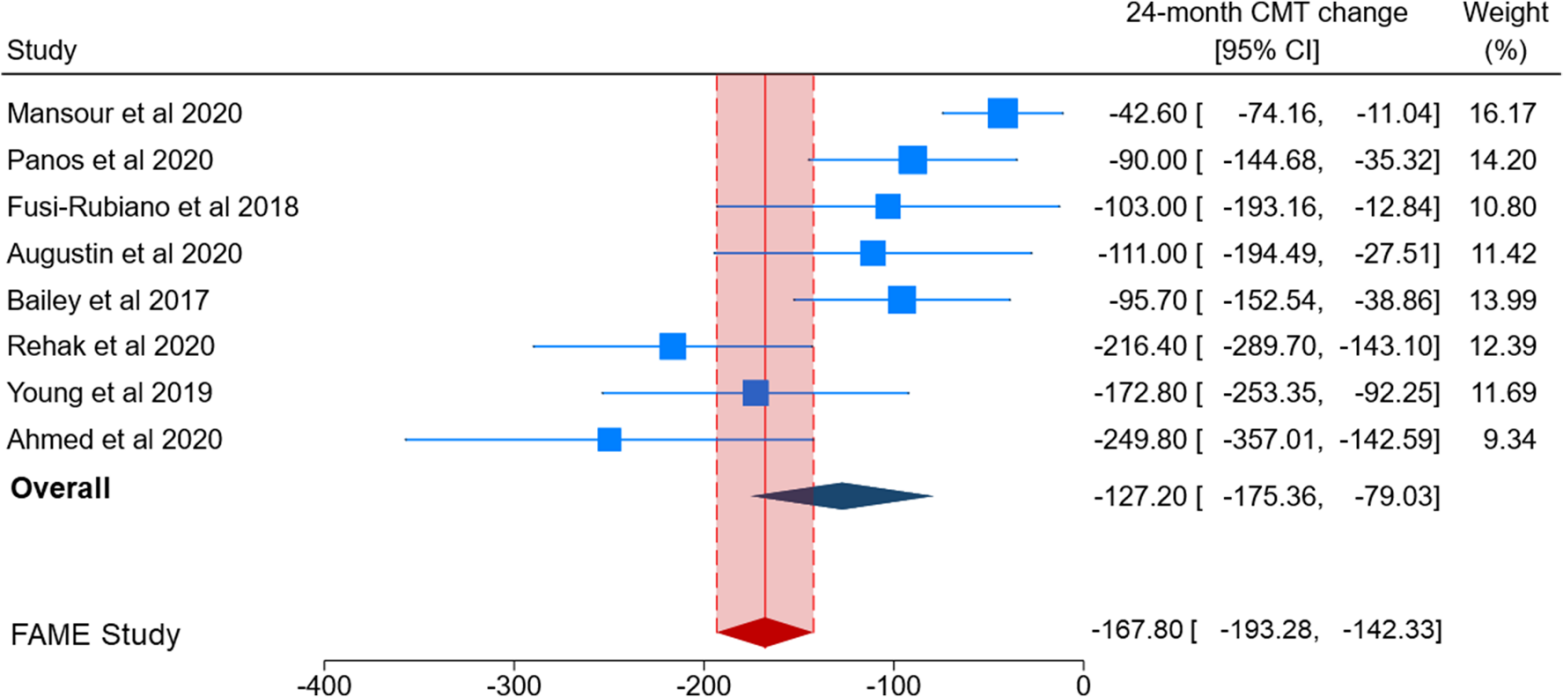
A forest plot showing 24-month central macular thickness (CMT) change, reported as Mean Difference (MD) with 95% confidence interval (CI), in real-world studies and 24-month foveal thickness change in the FAME study.

**Figure 5:**
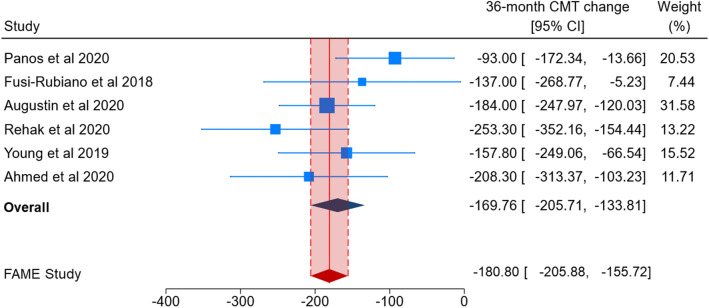
A forest plot showing 36-month central macular thickness (CMT) change, reported as Mean Difference (MD) with 95% confidence interval (CI), in real-world studies and 36-month foveal thickness change in the FAME study.

Figures 4 and 5 show the comparison of CMT change obtained by pooling real-world studies with that reported by the FAME study. The 0.2 µg/day FAc implant yielded a significantly reduced CMT at the 24-month follow-up in eight real-world studies (MD = −127.20; 95% CI = −175.36 to −79.03; Fig. 4). Because of significant heterogeneity across studies (*p* < 0.01 for Q-statistics and I^2^ = 78.7%), the random effect model was applied. A similar result was obtained by pooling six real-world studies reporting CMT change at the 36-month follow-up (MD = −169.76; 95% CI − 205.71 to − 133.81; Fig. 5), with a reduced heterogeneity across studies (*p* = 0.02 for Q-statistics and I^2^ = 31.9%). At the 24-month follow-up, most individual effect sizes from real-world studies did not fall within the 95% CIs reported by the FAME study. In line with this result the pooled estimate of real-world studies only partly overlapped that from the FAME study (Fig. 4). Results became more comparable at the 36-months of follow-up (Fig. 5).

### Supplementary intravitreal therapy and adverse events

**Figure 6:**
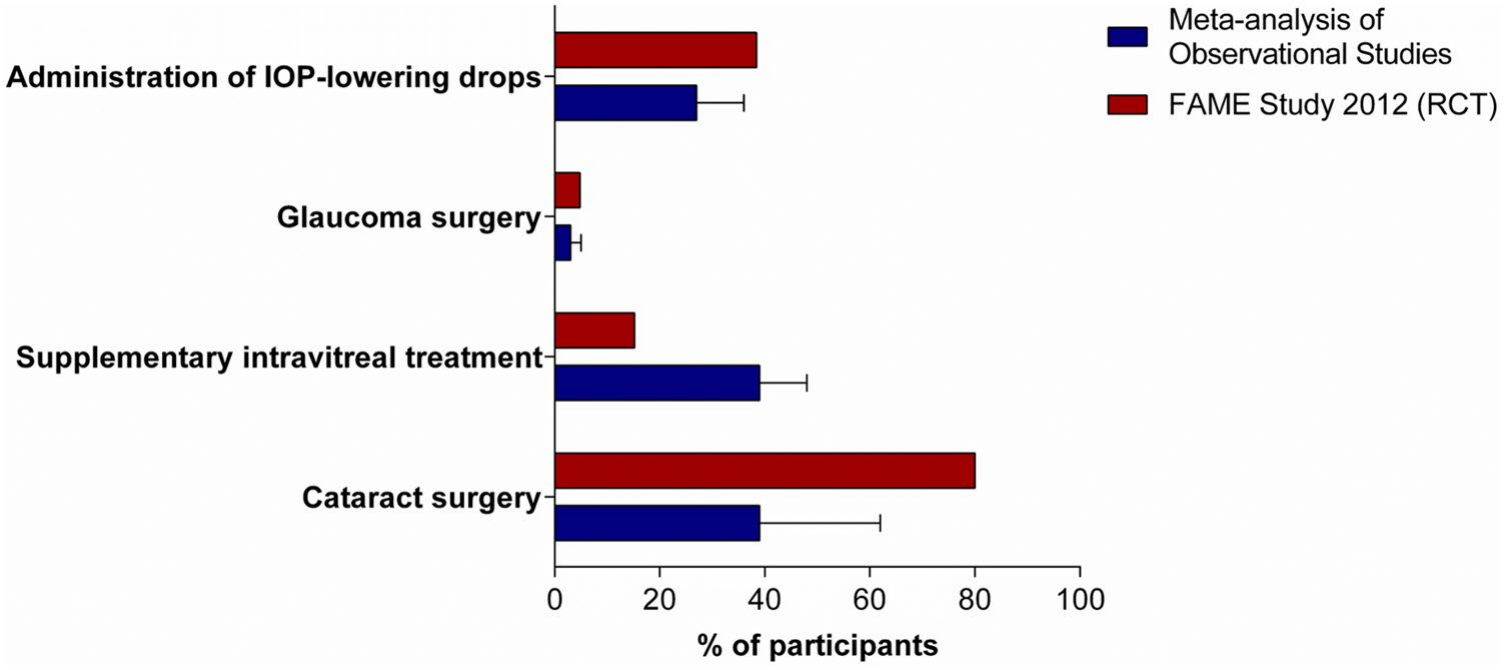
Pooled estimates of rates of eyes requiring cataract surgery, supplementary intravitreal treatment, intraocular pressure (IOP) lowering drops, glaucoma surgery. Blue histograms refer to pooled estimates from real-world studies; red histograms refer to rates from the 36-month FAME study.

We next pooled the proportion of eyes receiving supplementary intravitreal therapy, cataract surgery, IOP lowering drops and glaucoma surgery reported by real-world studies (Fig. 6). Specifically, the pooled proportions of eyes receiving cataract surgery, IOP lowering drops and glaucoma surgery were 39% (95% CI 18–62%), 27% (95% CI 19–36%) and 3% (95% CI 1–5%), respectively. These were all lower values than those reported by the FAME study (80%, 38.4% and 4.8%, respectively). By contrast, pooled estimate of eyes receiving supplementary intravitreal therapy was 39% (95% CI 31–48%), a higher value than the 15.2% reported by the FAME study.

## Discussion

The present meta-analysis explored for the first time real-world outcomes of 0.2 µg/day FAc intravitreal implant for chronic DMO, primarily showing that long-term visual improvement is comparable with the FAME study.

The FAME study reported the results of two parallel phase III randomized controlled trials comparing three different groups, namely the 0.2 µg/day FAc intravitreal implant, the 0.5 µg/day FAc intravitreal implant and a sham. These trials enrolled a total of 953 patients with chronic DMO and evaluated 36-month efficacy and safety of FAc implants. The FAME outcomes allowed the 0.2 µg/day FAc intravitreal implant to be licensed for the treatment of chronic DMO.

Randomized clinical trials have a primary role in evidence-based practice since the highest level of evidence is based on these type of trials^18^. Phase III trials investigate efficacy and safety of a drug and the results of these trials may allow drug licensing^19^. Phase IV trials and post-marketing studies evaluate the effectiveness and safety of the drug during the post-commercialization phase in a real-world setting^8,19^. Real-world studies report findings produced outside the context of RCTs. As such they are a reliable indicator of what should be expected in clinical practice^20^. These studies are of great value because clinical practice could fail to reach the same results obtained in RCTs. This is particularly relevant when it comes to chronic diseases that require continuous and intensive treatment, such as DMO.

A very recent real-world study including more than 28 thousand eyes with DMO demonstrated that real-world outcomes of intravitreal anti-VEGF therapy are worse than those of RCTs, irrespective of the anti-VEGF agent^4^. In real-world practice, patients received a mean of 6.3, 6.3 and 6.7 injections of bevacizumab, aflibercept and ranibizumab, respectively, over 1 year^4^, while these figures were 9.7, 9.2 and 9.4, respectively, in the DRCR.net Protocol T trial^21^. In real-world practice, 1 year visual gain was + 4.5, + 4.3 and + 3.4 letters for bevacizumab, aflibercept and ranibizumab, respectively^4^, while DRCR.net Protocol T reported a visual improvement of + 9.7, + 13.3 and + 11.2 letters, respectively, at 1 year^21^. Undertreatment mainly accounts for worse visual outcomes. This highlights unmet needs related to the burden of intravitreal anti-VEGF therapy, which currently represents the first-line treatment for DMO^4^.

The injection burden could be reduced by using sustained-release corticosteroid intravitreal implants, which present a long-lasting action as well as a proved clinical effectiveness for DMO treatment^22,23^.

A real-world study of 128 eyes on the use of the intravitreal 0.7 mg dexamethasone implant for DMO^24^ reported even better outcomes compared to the MEAD trial^25^: 25% of eyes achieved a 15-letter gain at 36 months^24^ versus 22% recorded in the MEAD trial^25^; a mean of 3.6 implants were administered over 36 months^24^ versus 4.1 recorded in the MEAD trial^25^.

Our meta-analysis demonstrated a visual gain of 4.52 letters and 7.89 letters at 24 months and 36 months, respectively, in agreement with a 24-month gain of 4.40 letters and a 36-month gain of 8.10 letters reported by the FAME study^6,7^. The fact that real-world evidence confirmed the visual improvement demonstrated by the RCT could be related to the long-term efficacy of the insert. It requires less frequent follow-ups and treatments compared with anti-VEGF. Thus, there is less chance of undertreatment. Our results confirmed the FAME finding of a better visual gain at 36 months compared with 24 months^6^. The authors speculated that such long-term benefits could be related to a trophic effect secondary to adequate control of inflammation^6^.

As concerns central retinal thickness, this meta-analysis yielded a CMT reduction of 127 µm and 170 µm at 24 months and 36 months, respectively. The 24-month result was characterized by higher heterogeneity and was less similar to the FAME finding compared to 36 months^6,7^. This comparison has to be considered cautiously because our outcome was mean CMT change, while the FAME study evaluated mean change in foveal thickness, defined as center point thickness, which is assumed to be the mean thickness at the crossing point of the 6 radial scans^6,7^. An average difference of approximately 30 µm has been reported between the 1-mm diameter CMT and the central point thickness^26^. Additionally, the FAME study used a time-domain OCT, while almost all studies included in the present meta-analysis were based on spectral-domain OCT. However, when evaluating mean changes, these minor differences are averaged out.

Cataract progression and IOP rise have been reported as the main adverse events following intravitreal FAc implant and intravitreal steroid use in general^6,7,23^. The included studies defined IOP rise applying different threshold values. Therefore, we chose to investigate the rate of patients requiring IOP-lowering drops and glaucoma surgery, which are two reliable measures of this complication in clinical practice. The pooled estimate rate of phakic eyes having cataract surgery was 39% in the real-world studies. This is much lower compared to the 80% rate seen at the 36-month follow-up of the FAME study^6^. Likewise, rates of eyes receiving IOP-lowering drops and glaucoma surgery were lower in the real-world setting compared with the 36-month follow-up of the FAME study (27% vs 38.4% and 3% vs 4.8%, respectively)^6^. Possible reasons to explain this difference could be an underestimation of these events because of the shorter follow-up of the included real-world studies compared to the 36-month follow-up of the FAME study as well as other biases and confounders related to a real-world setting. In particular, in clinical practice the FAc insert is often administered after treatment with other intravitreal steroids, such as a dexamethasone implant, which could help to select non-steroid responders. Moreover, the dexamethasone intravitreal implant showed a lower real-world^24^ rate of IOP rise compared with those of the MEAD trial^25^ (IOP ≥ 25 mmHg: 10.2% vs 32% at 36 months). Similarly, rates of cataract surgery following dexamethasone intravitreal implant were lower in real-world practice^24^ compared with the MEAD trial^25^ (47% vs 59%). Cataract surgery was associated with better outcomes when performed at the same time as intravitreal dexamethasone implant administration because of a reduction of postoperative inflammation^27^. This could be assumed to be valid also for the FAc implant.

The main advantage of the FAc intravitreal implant is long-term efficacy and, as a consequence, a reduced treatment frequency. In turn, this could result in a reduction of complications related to intensive anti-VEGF intravitreal injections. These include endophthalmitis with repeated intravitreal injections as well as a theoretical risk of death and cardiovascular events^28,29^. However, increased risk of mortality and cardiovascular events associated with intravitreal anti-VEGF therapy has not been demonstrated by registered clinical trials^30^. Meta-analysis studies have also shown no higher risk overall^28,31^, raising only a possible warning signal of increased risk in subjects with the highest-level of exposure (i.e. high risk diabetic patients receiving long-term intensive intravitreal anti-VEGF therapy)^28,30^.

Our results showed that 39% of eyes treated with the FAc implant in real-world practice received additional intravitreal therapy for DMO treatment, much greater than 15.2% reported by the 36-month FAME study^6^. Even if these two figures cannot be directly compared given the difference in methodology and design between real-world studies and RCTs, our analysis suggests that more than one third of patients treated with a FAc insert for chronic DMO could require an additional intravitreal therapy. It would have been informative to know how many treatments and with which frequency they were given in clinical practice, but such analyses were not conducted because of lack of evidence. This discrepancy between real-world practice and RCT could be explained by the fact that the FAME study was conducted in the period 2007–2010^7^, when the treatment of DMO was mainly performed with macular laser or off-label steroids. At that time, intravitreal anti-VEGF therapy was licensed for age-related macular degeneration, but not yet approved and scarcely used for DMO treatment^32^. In the FAME study, intravitreal anti-VEGF and triamcinolone were not deemed as allowable rescue treatment and were administered only in cases not experiencing any improvement^7^. Furthermore, the FAME study was based on time domain OCT imaging^7^, while most of the included real-world studies adopted spectral domain OCT imaging. All these factors probably contributed to the lower percentage of patients receiving additional treatment in the FAME study.

Additionally, 13% of patients enrolled in the FAME study received an additional 0.2 µg/day FAc implant during the follow-up period and this additional therapy could have reduced the need for other intravitreal agents. Conversely, only three real-world studies recorded the use of additional 0.2 µg/day FAc implants, with few patients (1–8.6%) having such a retreatment during the follow-up period.

This study had the following limitations. First, we conducted a meta-analysis of real-world studies, which, by definition, have different designs compared with RCTs. Therefore, no formal analysis could statistically compare real-world findings with RCT findings, but visual comparison of pooled estimates and 95% CIs with those obtained by RCT allowed us to assess whether real world outcomes matched those obtained with the RCT or not. Furthermore, while BCVA and CMT outcomes were reported by the included studies at the different time points of interest, namely 24 and 36 months, this was not the case for proportions of eyes receiving cataract surgery, IOP-lowering drops, glaucoma surgery and additional intravitreal therapy. All these proportions were provided throughout the study follow-up, which differed among the included studies. Nonetheless, in all cases mean follow-up exceeded 12 months. Ultimately, included studies might have been influenced by different clinical variables due to their real-world setting and bias could have been introduced. However, all meta-analyses were characterized by low heterogeneity across studies, except the one on the 24-month CMT. Included studies were deemed as low-to-moderate risk of bias. Funnel plots inspection revealed no significant risk of publication bias. All these support a good quality level of evidence. Moreover, a meta-analysis has more accurate confidence and higher power than a single report^33,34^.

In conclusion, our study revealed favorable outcomes in terms of visual improvement and macular thickness reduction following an intravitreal FAc implant for chronic DMO, which is in line with the findings reported by the FAME study. While the pooled proportion of cataract surgery and eyes experiencing requiring-treatment IOP rise are not concerning, the pooled estimate rate of eyes requiring additional intravitreal therapy is significant and further studies are warranted to better investigate this issue.

## Materials and methods

### Search method

This study was based on the Preferred Reporting Items for Systematic Reviews and Meta-Analyses (PRISMA) guidelines (PRISMA checklist available as Supplementary Table S2 online).

Systematic search of studies on FAc intreavitreal implant use for chronic DMO was conducted on Pubmed, Embase and Medline databases, from their inception up to 16th October 2020. The search strategy was performed including the terms ‘fluocinolone acetonide’, ‘diabetic macular edema’, ‘diabetic macular oedema’, ‘macular edema’, ‘macular oedema’, ‘diabetic retinopathy’, connected by and/or in various combinations. Reference lists of included studies and potentially eligible studies were also screened.

### Eligibility criteria

The following inclusion criteria had to be meet: (1) to report on the use of 0.2 µg/day FAc intravitreal implant for chronic DMO; (2) to report outcomes at 24-month follow-up or longer; (3) to report data on the primary outcome of this meta-analysis; (4) to include a minimum of 10 patients for the primary outcome measure of this meta-analysis. No restriction on study design was imposed. Only articles published in peer-reviewed journals and in English were considered. Abstracts and conference posters were excluded. Reports including only vitrectomized eyes were excluded as well. Real-world studies were defined as those reporting data collected outside the context of RCTs^20^.

The primary outcome of the present study was the mean change in best corrected visual acuity (BCVA) following FAc intravitreal implant at 24 months. Secondary outcomes included BCVA change at 36 months, the mean change in optical coherence tomography (OCT) central retinal thickness (CMT), the rate of supplementary intravitreal therapy, and the rate of adverse events, such as cataract surgery, rates of eyes requiring IOP lowering drops and glaucoma surgery. Central macular thickness was the average value of the fovea-centered area with 1 mm diameter^35^. Supplementary intravitreal therapy indicated any intravitreal therapy administered during the follow-up after FAc intravitreal implant, except repeated FAc intravitreal implant.

### Data collection and quality assessment

Two investigators (M.F. and A.L.) independently screened titles and abstracts of all identified articles, applying eligibility criteria. A full-text review was conducted on all potentially eligible studies to evaluate if inclusion/exclusion criteria were completely fulfilled. A third investigator (T.A.) was consulted in case of disagreement to achieve consensus. When additional information or clarifications were necessary for eligibility assessment or data extraction, the authors of the study were contacted. Two investigators (M.F. and A.L.) independently analyzed and collected data from the included studies. Data extraction included the following items: first author, study year, design and location, mean age, number of patients, follow-up, BCVA change, CMT change, proportions of eyes receiving supplementary intravitreal therapy, cataract surgery, IOP lowering drops and glaucoma surgery.

Risk of bias was evaluated by using the Cochrane collaboration tool^36^ and the Methodological item for non-randomized studies (MINORS) scale^37^ for RCTs and non-randomized studies, respectively. A MINORS score ≥ 9 was considered as low-to-moderate risk of bias.

### Statistical analysis

We first meta-analyzed effect sizes for primary and secondary outcomes obtained from real-world studies. Specifically, primary outcome was BCVA change at the 24-month follow-up, reported as mean differences (MDs) between post-treatment and baseline values and their 95% Confidence Interval (95% CI). Similarly, 36-month BCVA change and CMT change were reported as MD with 95% CIs. Further secondary outcomes included the proportion of eyes receiving supplementary intravitreal therapy, cataract surgery (i.e. exclusively among phakic eyes), IOP lowering drops and glaucoma surgery. For each individual study, the score confidence intervals were constructed and proportions were pooled using the Metaprop command on Stata (version 16)^38^. Heterogeneity across studies was tested using the Q-statistics and the I^2^ index. A fixed effect model was applied in the absence of significant heterogeneity, while a random effect model with the DerSimonian-Laird method was applied if *p* value for Q-statistics < 0.1 and I^2^ > 50%. The extent of publication bias was explored by Funnel plots and tested using Egger’s test.

Pooled effect sizes with their 95%CI were compared with those obtained by the RCT.

All the statistical analyses were carried out on STATA (version 16) with significance level α < 0.05 if not otherwise stated.

### Ethical approval and informed consent

Since this is a systematic review, ethical approval and informed consent are not required.

## Supplementary Information

Supplementary Informations.
