# Supplementary material for: Fluocinolone acetonide vitreous insert for chronic diabetic macular oedema: a systematic review with meta-analysis of real-world experience
Source: Sci Rep. 2021 Feb 26;11:4800. doi: 10.1038/s41598-021-84362-y (PMC7910468; doi:10.1038/s41598-021-84362-y)

Supplementary Information

Fluocinolone acetonide vitreous insert for chronic diabetic macular oedema: a systematic review with meta-analysis of real world experience.

Matteo Fallico[1*], Andrea Maugeri[2*], Andrew Lotery[3], Antonio Longo[1], Vincenza Bonfiglio[4], Andrea Russo[1], Teresio Avitabile[1], Claudio Furino[5], Gilda Cennamo[6], Martina Barchitta[2], Antonella Agodi[2], Paola Marolo[7], Luca Ventre[7], Paolo Caselgrandi[7], Michele Reibaldi[7]

[1]*Department of Ophthalmology, University of Catania, 95123 Catania, Italy;*
[2]*Department of Medical and Surgical Sciences and Advanced Technologies "GF Ingrassia", University of Catania, 95123 Catania, Italy;*
[3]*Faculty of Medicine, University of Southampton, UK;*
[4]*Department of Experimental Biomedicine and Clinical Neuroscience, Ophthalmology Section, University of Palermo, 90127 Palermo, Italy;*
[5]*Department of Ophthalmology, University of Bari, 70124 Bari, Italy;*
[6]*Department of Public Health, University of Naples Federico II, 80131 Naples, Italy*
[7]*Department of Surgical Sciences, Eye Clinic Section, University of Turin, 10122 Turin, Italy*

* Dr Fallico and Dr Maugeri contributed equally and should therefore be regarded as equivalent first authors and co-corresponding authors.

Corresponding authors:
Matteo Fallico, MD
Department of Ophthalmology, University of Catania, Via S. Sofia 78, 95123, Catania, Italy
email: matteofallico@hotmail.com
phone number: +390953781213
Andrea Maugeri, PhD
Department of Medical and Surgical Sciences and Advanced Technologies "GF Ingrassia", University of Catania, Via S. Sofia 87, 95123 Catania, Italy
email: andrea.maugeri@unict.it
phone number: +390953782076

Table S1: MINORS scores of non-randomized studies.

Author, year	clearly stated aim	consecutive patients	Prospective data collection	Endpoints appropriate to the aim of the study	Unbiased assessment of the study endpoint	Follow-up period appropriate to the aim of the study	Loss to follow up less than 5%	Prospective calculation of the study size	An adequate control group	Contemporary groups	Baseline equivalence of groups	Adequate statistical analyses	Total
Mansour et al 2020	2	0	2	2	2	2	2	NA	NA	NA	NA	2	14/16
Panos et al 2020	2	0	0	2	2	2	2	NA	NA	NA	NA	2	12/16
Augustin et al 2020	2	0	0	2	2	2	1	NA	NA	NA	NA	2	11/16
Rehak et al 2020	2	0	0	2	2	2	1	NA	NA	NA	NA	2	11/16
Ahmed et al 2020	2	0	0	2	2	2	1	NA	NA	NA	NA	2	11/16
Young et al 2019	2	2	0	2	2	2	1	NA	NA	NA	NA	1	12/16
Chakravarthy et al 2018	2	0	2	2	2	2	1	NA	NA	NA	NA	2	13/16
Fusi-Rubiano et al 2018	2	0	0	2	2	2	1	NA	NA	NA	NA	2	11/16
Bailey et al 2017	2	0	0	2	2	2	1	NA	NA	NA	NA	2	11/16

Footnote: MINORS= Methodological item for non-randomized studies; 0= not reported; 1= reported, but inadequate; 2= reported and adequate; NA: not applicable.

Table S2: PRISMA checklist.

Section/topic	#	Checklist item	Reported on page #
**TITLE**			1
Title	1	Identify the report as a systematic review, meta-analysis, or both.	1
**ABSTRACT**			2
Structured summary	2	Provide a structured summary including, as applicable: background; objectives; data sources; study eligibility criteria, participants, and interventions; study appraisal and synthesis methods; results; limitations; conclusions and implications of key findings; systematic review registration number.	2
**INTRODUCTION**			3
Rationale	3	Describe the rationale for the review in the context of what is already known.	3
Objectives	4	Provide an explicit statement of questions being addressed with reference to participants, interventions, comparisons, outcomes, and study design (PICOS).	3
**METHODS**			11-14
Protocol and registration	5	Indicate if a review protocol exists, if and where it can be accessed (e.g., Web address), and, if available, provide registration information including registration number.	-
Eligibility criteria	6	Specify study characteristics (e.g., PICOS, length of follow-up) and report characteristics (e.g., years considered, language, publication status) used as criteria for eligibility, giving rationale.	12
Information sources	7	Describe all information sources (e.g., databases with dates of coverage, contact with study authors to identify additional studies) in the search and date last searched.	11
Search	8	Present full electronic search strategy for at least one database, including any limits used, such that it could be repeated.	11
Study selection	9	State the process for selecting studies (i.e., screening, eligibility, included in systematic review, and, if applicable, included in the meta-analysis).	12
Data collection process	10	Describe method of data extraction from reports (e.g., piloted forms, independently, in duplicate) and any processes for obtaining and confirming data from investigators.	13
Data items	11	List and define all variables for which data were sought (e.g., PICOS, funding sources) and any assumptions and simplifications made.	13-14
Risk of bias in individual studies	12	Describe methods used for assessing risk of bias of individual studies (including specification of whether this was done at the study or outcome level), and how this information is to be used in any data synthesis.	14
Summary measures	13	State the principal summary measures (e.g., risk ratio, difference in means).	14

Synthesis of results	14	Describe the methods of handling data and combining results of studies, if done, including measures of consistency (e.g., I^2) for each meta-analysis.	14
**Section/topic**	**#**	**Checklist item**	**Reported on page #**
Risk of bias across studies	15	Specify any assessment of risk of bias that may affect the cumulative evidence (e.g., publication bias, selective reporting within studies).	14
Additional analyses	16	Describe methods of additional analyses (e.g., sensitivity or subgroup analyses, meta-regression), if done, indicating which were pre-specified.	14
**RESULTS**			4-6
Study selection	17	Give numbers of studies screened, assessed for eligibility, and included in the review, with reasons for exclusions at each stage, ideally with a flow diagram.	4
Study characteristics	18	For each study, present characteristics for which data were extracted (e.g., study size, PICOS, follow-up period) and provide the citations.	4-5
Risk of bias within studies	19	Present data on risk of bias of each study and, if available, any outcome level assessment (see item 12).	5
Results of individual studies	20	For all outcomes considered (benefits or harms), present, for each study: (a) simple summary data for each intervention group (b) effect estimates and confidence intervals, ideally with a forest plot.	4-5
Synthesis of results	21	Present results of each meta-analysis done, including confidence intervals and measures of consistency.	5-6
Risk of bias across studies	22	Present results of any assessment of risk of bias across studies (see Item 15).	5
Additional analysis	23	Give results of additional analyses, if done (e.g., sensitivity or subgroup analyses, meta-regression [see Item 16]).	5-6
**DISCUSSION**			7-11
Summary of evidence	24	Summarize the main findings including the strength of evidence for each main outcome; consider their relevance to key groups (e.g., healthcare providers, users, and policy makers).	7-10
Limitations	25	Discuss limitations at study and outcome level (e.g., risk of bias), and at review-level (e.g., incomplete retrieval of identified research, reporting bias).	10-11
Conclusions	26	Provide a general interpretation of the results in the context of other evidence, and implications for future research.	11
**FUNDING**			17
Funding	27	Describe sources of funding for the systematic review and other support (e.g., supply of data); role of funders for the systematic review.	17

From: Moher D, Liberati A, Tetzlaff J, Altman DG, The PRISMA Group (2009). Preferred Reporting Items for Systematic Reviews and Meta-Analyses: The PRISMA Statement. PLoS Med 6(7): e1000097. doi:10.1371/journal.pmed1000097

Figure S1: Funnel plot for 24-month best corrected visual acuity change

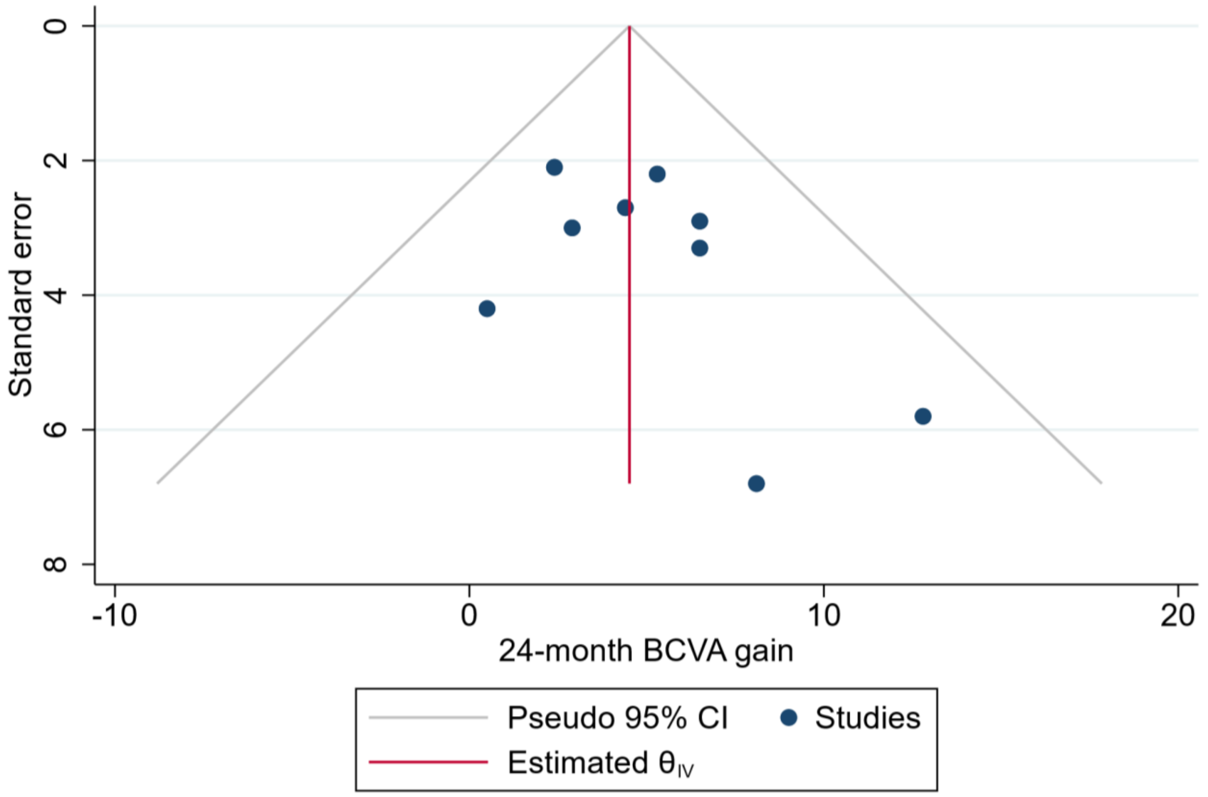

Figure S2: Funnel plot for 36-month best corrected visual acuity change

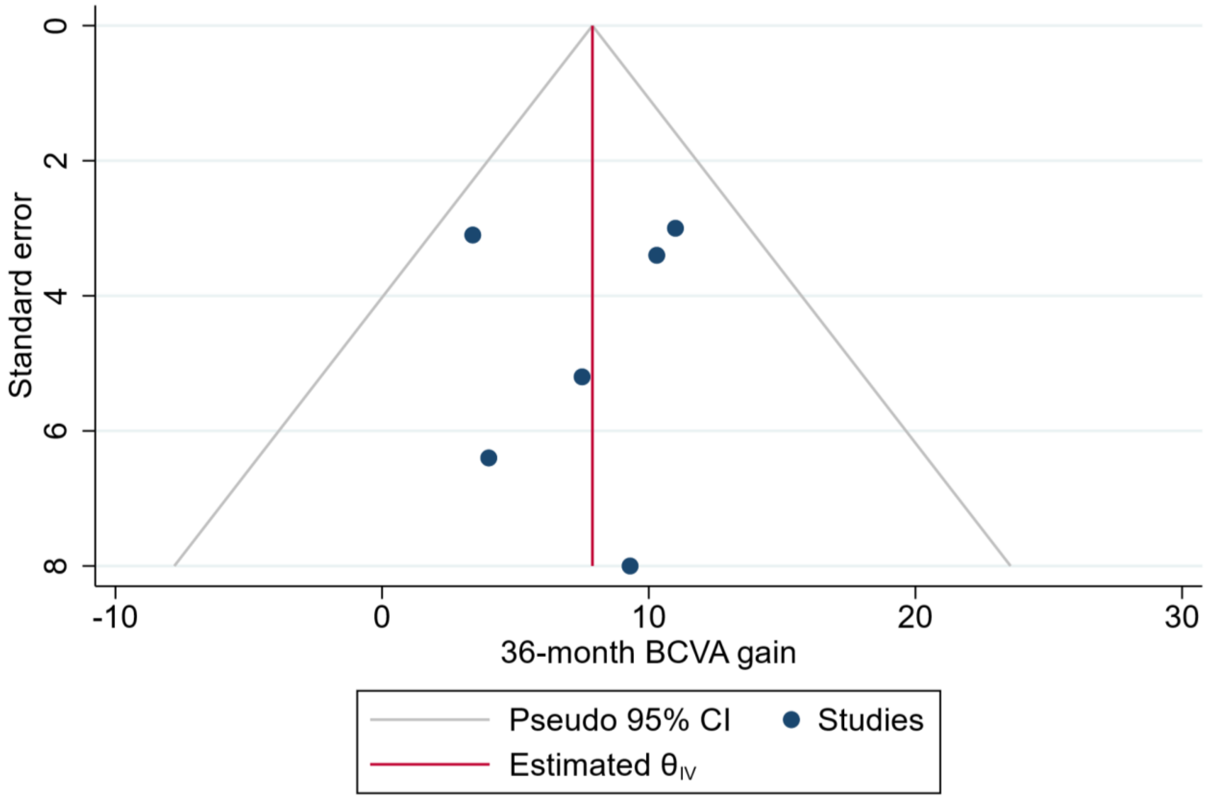

Figure S3: Funnel plot for 24-month central macular thickness change

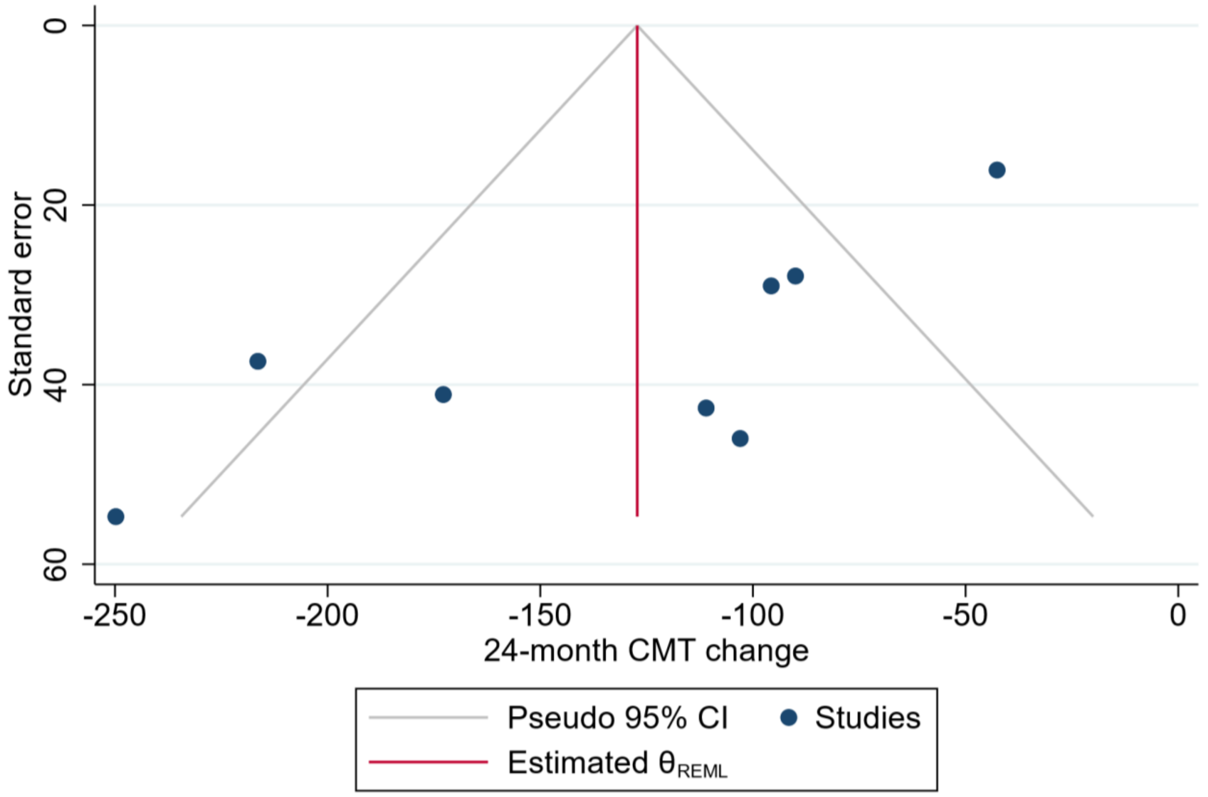

Figure S4: Funnel plot for 36-month central macular thickness change

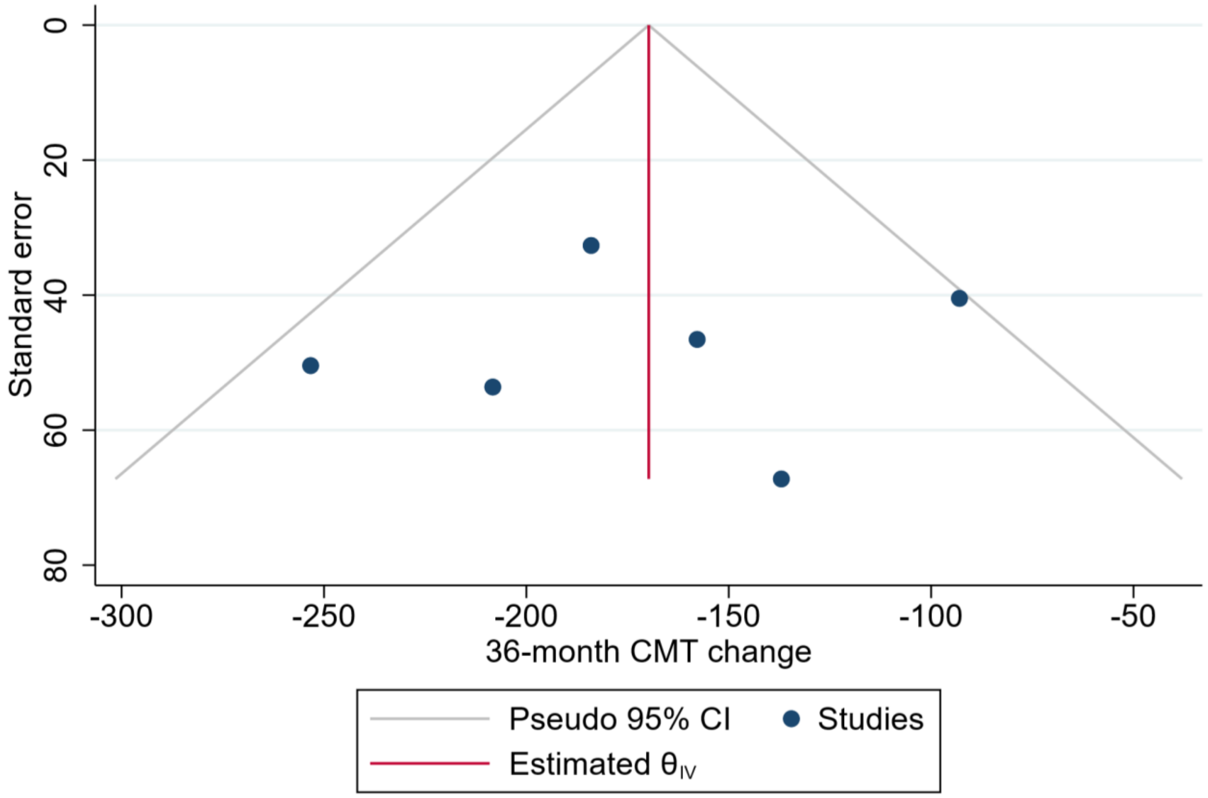

Supplement: Supplementary file 1 — Supplementary Informations. [file 41598_2021_84362_MOESM1_ESM.pdf]
